# Enhancing Basketball Game Outcome Prediction through Fused Graph Convolutional Networks and Random Forest Algorithm

**DOI:** 10.3390/e25050765

**Published:** 2023-05-08

**Authors:** Kai Zhao, Chunjie Du, Guangxin Tan

**Affiliations:** School of Physical Education and Sports Science, South China Normal University, Guangzhou 510006, China; 2022021150@m.scnu.edu.cn (K.Z.); duchunjie@m.scnu.edu.cn (C.D.)

**Keywords:** unstructured data, graph convolutional network, game outcome prediction, features extraction, random forest

## Abstract

Basketball is a popular sport worldwide, and many researchers have utilized various machine learning models to predict the outcome of basketball games. However, prior research has primarily focused on traditional machine learning models. Furthermore, models that rely on vector inputs tend to ignore the intricate interactions between teams and the spatial structure of the league. Therefore, this study aimed to apply graph neural networks to basketball game outcome prediction, by transforming structured data into unstructured graphs, to represent the interactions between teams in the 2012–2018 NBA season dataset. Initially, the study used a homogeneous network and undirected graph to build a team representation graph. The constructed graph was fed into a graph convolutional network, which yielded an average success rate of 66.90% in predicting the outcome of games. To improve the prediction success rate, feature extraction based on the random forest algorithm was combined with the model. The fused model yielded the best results, and the prediction accuracy was improved to 71.54%. Additionally, the study compared the results of the developed model with previous studies and the baseline model. Our proposed method considers the spatial structure of teams and the interaction between teams, resulting in superior performance in basketball game outcome prediction. The results of this study provide valuable insights for basketball performance prediction research.

## 1. Introduction

Machine learning (ML) is an interdisciplinary field that combines computer science, statistics, and other disciplines to develop predictive models that imitate certain aspects of human thinking. Accurate prediction is essential for various industries, including policy-making, risk prevention, resource management, and economic and social development. In the sports industry, prediction models are increasingly used by coaches, players, and companies to improve competitiveness and profits. For example, accurate predictions can inform sales planning, investment decisions, training programs, tactical choices, and injury prevention strategies [1,2,3].

Sports industries have grown rapidly in recent decades, driven by economic, technological, and social developments. Sports markets generate significant value and revenue, such as through sports betting, venue management, and broadcast management, as exemplified by the 2022 FIFA World Cup in Qatar. With the rise in popularity of sports, there has been growing interest in predicting sports outcomes [4,5,6]. Among all sports, the National Basketball Association (NBA) in the United States is one of the most influential basketball leagues, generating billions of dollars in revenue. Winning games, gaining advantages, and maintaining team performance are crucial goals for competitive organizations such as the NBA. To achieve these goals, coaches and team administrators analyze and predict future team and player performance, and adjust team lineups and tactics accordingly.

While sports outcome prediction has received widespread attention [7,8,9,10,11,12], the existing research has mainly focused on basketball, particularly NBA games, using data mining methods or traditional machine learning models [13,14,15,16,17,18,19,20]. However, there has been less research on applying graph neural networks (GNN) to predict basketball game outcomes. This paper will use the NBA as an example to build a team representation graph. To increase the model’s utility in different sports and basketball leagues, we will use a homogeneous network and undirected graph to predict winning and losing outcomes in basketball games.

In this paper, we propose a basketball game outcome prediction method based on graph convolutional networks. The GCN methodology has been widely applied to various practical fields, such as computer vision, natural language processing, traffic, recommender systems, and chemistry [21]. We chose GCN as our prediction model, due to its ability to consider the influence of competitors, gain insight into team relationships, and its applicability to different sports and basketball leagues.

The proposed model was applied to the NBA dataset from 2012 to 2018. We transformed traditional structured data into unstructured graph data to represent NBA teams spatially and used the random forest (RF) algorithm for feature extraction. We then performed a GCN-based game outcome prediction analysis, using the constructed graph. Our aim is to stimulate information transfer between teams, deepen their understanding of their strengths, and improve future performance based on predictions.

This paper contributes to the literature in several ways. First, we apply GCN to the basketball game prediction problem, which, to the best of our knowledge, has not been done before. Second, we convert NBA game data into graph structure data, which allows us to understand the advantages of each team in the NBA and use it for game result prediction analysis. Third, we use RF and other methods to extract features from the NBA dataset and use the GCN model to predict the game outcome, resulting in a better prediction accuracy than previous studies.

Section 2 reviews the relevant literature, while Section 3 briefly describes the overall flow of our study, explains the methodology used in the study, and presents the graph structure of the NBA. In Section 4, we introduce the dataset, apply the proposed model and algorithm to the NBA dataset, and compare and evaluate it with the baseline model. Finally, Section 5 discusses and concludes the paper.

## 2. Related Work

It is important to note that this paper proposes a graph convolutional network (GCN) prediction model for basketball games, which is specifically applied to the NBA dataset. Therefore, the relevant literature for this study mainly focused on two aspects: basketball game outcome prediction and the GNN methodology, as well as sports outcome prediction.

### 2.1. Basketball Game Outcome Prediction

In this section, we will examine the data sets used in previous studies for predicting basketball game outcomes, the number of features used in each study, the most successful algorithm employed, and the corresponding success rates achieved.

In recent years, several studies have been conducted to predict the outcomes of basketball games using machine learning algorithms. Loeffelholz et al. [22] used neural networks to model the NBA 2007–2008 season and found that the most effective method was the feed-forward neural network (FFNN), with a success rate of 74.33%. Similarly, Zdravevski and Kulakov [23] predicted two consecutive NBA seasons using algorithms in Weka, with logistic regression being the most effective method, with a success rate of 72.78%. On the other hand, Miljkovic et al. [24] predicted the NBA 2009–2010 season games using data mining and found that the most efficient method was naive Bayes, with a success rate of 67%.

Cao [25] used machine learning algorithms to build a model for predicting NBA game outcomes and found that simple logistic regression was the most effective method, with a success rate of 69.67%. Lin, J. et al. [26] attempted to determine the winners of NBA 1991–1998 season games using random forests and achieved a 65% success rate. Tran, T. [27] predicted NBA games using matrix factorization, with an accuracy of 72.1%.

Li, Y. et al. [13] attempted to predict the games for the NBA 2011–2016 seasons using a data envelopment analysis methodology and tested with the 2015–2016 season, with a 73.95% accuracy rate. Horvat, T. et al. [17] used seven different machine learning models to predict basketball game outcomes for the NBA 2009–2018 season and found that k-nearest neighbors was the best method, with an accuracy of 60.01%. Li [28] conducted a study on modeling the NBA 2012–2018 season using machine learning classifiers. The study employed three different classifiers and found that linear regression following use of a least absolute shrinkage and selection operator (LASSO) was the most effective method, achieving a success rate of 67.24%. In another study conducted by Ozkan I A. [18], the outcomes of games from the 2015–2016 season of the Turkish Basketball Super League were estimated using a concurrent neuro-fuzzy system, with a 79.2% success rate.

ÇENE E. [29] explored the performance of seven different algorithms in predicting EuroLeague games for the 2016–2017 and 2020–2021 seasons. Their findings revealed that logistic regression, support vector machines (SVM), and artificial neural networks (ANN) were the most effective models, with an overall accuracy of approximately 84%.

Another study by Osken C and Onay C. [30] focused on identifying player types using k-means and c-means clustering, and using cluster memberships to train prediction models. Their approach achieved a prediction accuracy of 76% over a period of five NBA seasons.

Table 1 presents a comparison of previous studies that have attempted to predict basketball game outcomes. The table displays the datasets used in each study, the number of data points and features, the most successful algorithm used, and the corresponding success rates achieved.

### 2.2. GNN Methodology and Sport Outcome Prediction

The second category of relevant research focused on GNN, which is a cutting-edge method used for sports outcome prediction. This technique can be traced back to Aleksandra P. (2021) [31], who used the GNN model to predict the outcomes of soccer games. In the study, the author employed individual teams as nodes, past games as edges, and assigned different weights to the edges, to reflect recent games’ greater impact on the team performance. The model was trained on a soccer dataset of a league, with an accuracy of 52.31%. Similarly, Xenopoulos P. and Silva C. (2021) [32] used a graph-based representation of the state of the game as input to GNN to predict the outcome of NFL and CSGO games, resulting in a reduction of test set losses by 9% and 20%, respectively. Additionally, Mirzaei A. (2022) [33] utilized dynamic graph (specifically a spatiotemporal graph) representation learning to predict soccer games, using only the names of teams and players, achieving an estimation accuracy of 50.36%. Bisberg A J and Ferrara E. (2022) [34] modeled LoL (League of Legends) games with GCN, achieving an estimation accuracy of 61.9%. The training, validation, and test networks were LPL (League of Legends Pro League), LCK (LoL Champions Korea), and LCS (League of Legends Championship Series), respectively. Moreover, the utilization of GCN combined with RF has proven to be successful in various relevant cross-domain areas. For instance, Chen et al. [35] proposed a model that integrates random forest and Graph WaveNet to capture spatial dependencies and extract long-term dependencies from spatiotemporal data. The efficacy of this approach was demonstrated on real-world datasets for traffic flow and groundwater level prediction, resulting in improved performance. Similarly, in the field of activity recognition, Hu et al. [36] proposed a correlation coefficient-based method to generate a graph from motion signals, followed by random forest classification, resulting in a significantly high accuracy. Table 2 compares previous studies’ use of GNN to predict game outcomes, including the datasets, amount of data and features, the most successful models, and success rates.

Several researchers have developed GNN prediction models for various sports, but these models generally suffer from low accuracy. While some methods for predicting NBA game outcomes exist, few studies have applied GNN to basketball games. Thus, this paper proposes a new basketball game outcome prediction model and explores its application in the NBA.

In contrast to previous research, this study divides NBA games played between the 2012 and 2018 seasons into six datasets and extracts features using principal component analysis (PCA), least absolute shrinkage and selection operator (LASSO), and random forest (RF) models. The results are then predicted using GCN.

## 3. Methodology

**Figure 1:**
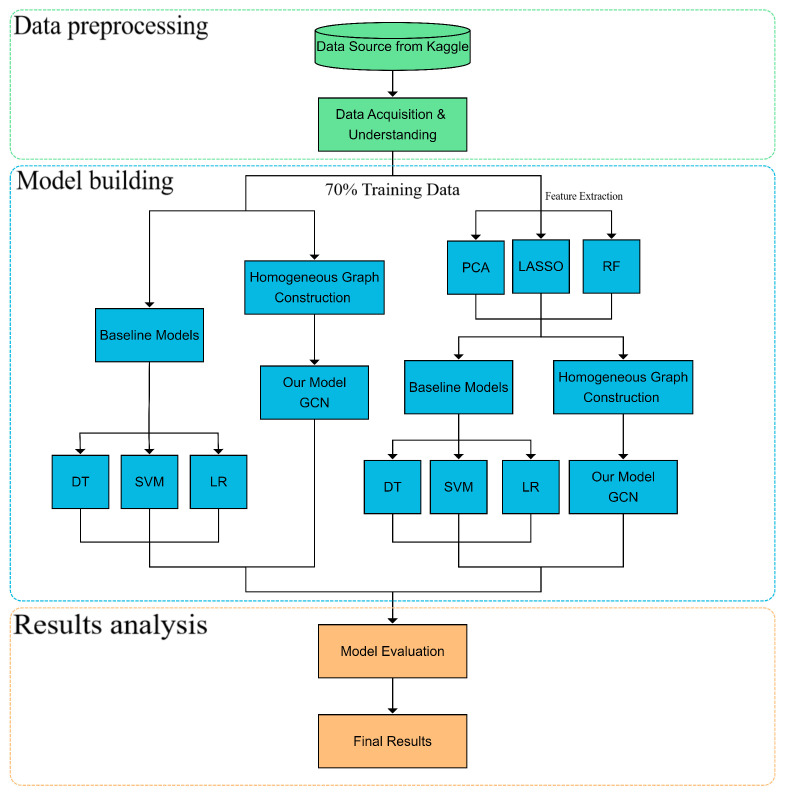
Basic flowchart of the study.

The following Figure 1 depicts an outline of the process of this paper. In the subsequent subsections, we will provide a more detailed explanation of each step.

### 3.1. Graph Networks Construction

The objective of this study was to develop a team structure in the NBA that can be used to predict game outcomes. To achieve this objective, we needed to create a graph that defines the nodes and their relationships. In our research, we employed a homogeneous graph that has only one node type and one relationship type. This type of graph provides a simplified representation of the graph data and can be used in other basketball leagues.

**Figure 2:**
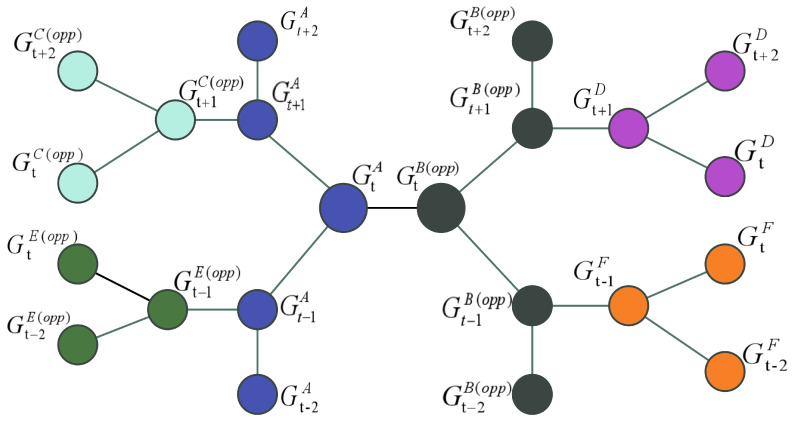
An illustration of the proposed homogeneous graph for sport outcome prediction.

An example of a homogeneous graph is presented in Figure 2. In this graph, each node represents a team and contains information about 44 features. Nodes are labeled as wins or losses for games, and the edges between the nodes represent recent games related to the teams. Specifically, at time t, team A is directly connected to opponent team B, at time t + 1, team A is directly connected to team C, and team B is directly connected to team D, and at time t − 1, team A is directly connected to team E, and team B is directly connected to team F. Moreover, each team is also connected to itself from its last game.

Since the structure of the graph is symmetric, the games are arranged in ascending chronological order for each team. Thus, one team in the graph is connected to its own last and next games. This structure enables us to consider the impact of recent games on the prediction of game outcomes. Additionally, the graph convolutional network (GCN) is able to learn the structure of the network built. Therefore, the prediction task can also be interpreted as a node classification task.

### 3.2. Principal Component Analysis

Principal component analysis (PCA) is a widely used statistical technique that aims to reduce the dimensionality of a dataset, while retaining the maximum amount of original variation. The underlying concept of PCA involves finding the directions, also known as “principal components”, in which the data varies the most, and projecting the data onto these components.

Specifically, PCA identifies the first principal component as the direction with maximum variability in the data, and the subsequent principal components correspond to the direction of maximum variability, after removing the earlier components. The number of principal components equals the number of variables in the original dataset.

Our first step in performing a PCA on the data was to standardize it by subtracting the mean of each variable from each data point and dividing it by the standard deviation of each variable. Next, we computed the covariance matrix, which provides information about the relationship between each pair of variables. Using the covariance matrix, we derived the eigenvalues and eigenvectors, which indicate the amount of variance explained by each principal component and the direction of the principal components, respectively. These principal components are created through linear combinations of the original variables and are sorted in descending order of their corresponding eigenvalues. Based on a predetermined number of components or the desired level of variance to be explained, we then selected the top k principal components that accounted for the most variance in the data. Finally, we projected the original data onto the principal components, to transform it into the new coordinate system defined by the principal components.

By selecting only the most important principal components, PCA reduces the number of variables required to describe the data, making the analysis more manageable and efficient. This can also lead to an improved accuracy and interpretability of the results.

### 3.3. Feature Extraction Based on the LASSO Algorithm

The LASSO algorithm is a widely used method for feature selection in machine learning. Its primary purpose is to extract a subset of important features from a large pool of available features. The algorithm operates by adding a penalty term to the cost function of a linear regression model. This penalty term encourages the coefficients of less important features to be reduced to zero. As a result, the LASSO algorithm produces a sparse model, where only the most significant features are retained.

The LASSO (least absolute shrinkage and selection operator) algorithm is a form of regularization technique, which is commonly used to reduce the complexity of a model by selecting the most relevant features, while setting the coefficients of less important features to zero.

The LASSO algorithm operates by adding a penalty term to the linear regression objective function, which encourages sparsity in the coefficients. The penalty term is the sum of the absolute values of the coefficients multiplied by a hyperparameter, known as the regularization parameter. By increasing the regularization parameter, the LASSO algorithm sets more coefficients to zero, effectively removing the corresponding features from the model.

### 3.4. Feature Extraction Based on the Random Forest Algorithm

Random forest is a widely used machine learning algorithm for classification and regression tasks, belonging to the ensemble learning category, where it aggregates predictions of multiple models to generate a final prediction. Random forest can also be employed for feature extraction, by identifying the most significant features within a dataset. The algorithm works by creating multiple decision trees, where each tree is trained on a random subset of data and features. The final prediction is then made by aggregating the results of all trees.

During the training of the model, random forest calculates the importance of each feature based on its contribution to the overall accuracy of the model. The features that have a higher contribution are assigned higher scores. By analyzing these scores, we can identify the most important features within the dataset. This process is beneficial when dealing with high-dimensional data or when we want to simplify the model without compromising its accuracy.

**Figure 3:**
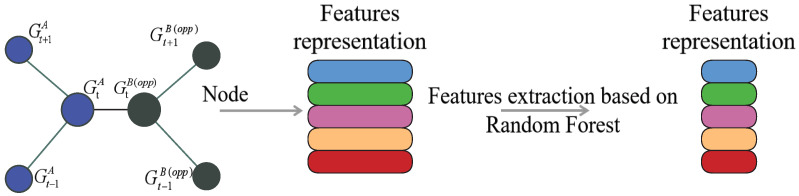
Schematic diagram of feature dimensionality reduction based on random forest.

In conclusion, random forest is a valuable tool for feature extraction and can assist in identifying the most relevant features within a dataset, which can be used to improve machine learning models. Figure 3 depicts the process of feature extraction using random forest.

### 3.5. Graph Convolutional Network

Graph convolution networks (GCN) are neural networks that process data represented by graph structures, making them an effective tool for analyzing and modeling complex data that cannot be modeled using traditional Euclidean space models. Unlike traditional convolutional neural networks that operate on images, GCN uses a convolution operator on the graph structure.

The formula for graph convolution is expressed as

(1) $$h_{i}^{\left(l+1\right)}=\sigma \left(\sum_{j\in N_{i}}\frac{1}{c_{ij}}W^{\left(l\right)}h_{j}^{\left(l\right)}\right)$$

Here, $h_{i}^{\left(l\right)}$ denotes the feature vector of node *i* at layer *l*, $W^{\left(l\right)}$ represents the weight matrix at layer *l*, $c_{ij}$ is a normalization constant, and σ is an activation function. $N_{i}$ represents the set of neighboring nodes to node *i*.

The aforementioned formula serves as the fundamental operation in GCN. The output feature vector of a node is a weighted sum of its neighboring nodes’ feature vectors, passed through a nonlinear activation function. This process is repeated for multiple layers, enabling GCN to learn hierarchical representations of the graph structure. More experimental details can be found in the following link: https://github.com/KaiZhao-Aike/nbagcn.git (accessed on 20 March 2023).

## 4. Experiment and Results

In this study, our goal was to accurately predict the outcomes of NBA games. We elaborate on the dataset, experimental procedures, and results in the subsequent subsections.

### 4.1. Datasets

Acquiring sufficient relevant data is a fundamental requirement for building effective prediction models. With the era of big data and rapid technological advancements in the sports industry, obtaining statistical information on sports has become easier. In this study, we utilized a dataset containing NBA statistics from 2012 to 2018 obtained from Kaggle, a data modeling and analysis competition platform that enables companies and researchers to explore machine learning. The dataset, submitted by Paul Rosetti, is called “NBA Enhanced Box Score and Standings (2012–2018)” [37]. The file 2012-18_teamBoxScore.csv, included in this dataset, contains basic and advanced data for each of the 82 games played by 30 NBA teams in each season from the 2012–2013 to the 2017–2018 seasons. It covers only the regular games of the NBA seasons during this period. The dataset consists of two rows for each game, representing the home team and the away team, with a total of 14,758 rows (excluding one game in the 2012–2013 season). To predict game outcomes using GCN, we divided the statistics for each season into separate datasets, resulting in six datasets in total. Table 3 provides the definitions of the 44 features used in this study. All the features used in our prediction model were calculated per team and per game. They were calculated by aggregating the statistics of the players who played in the game for each team. We calculated features such as “Team2p%” and “Team3p%” by computing the percentage of 2-point and 3-point shots made by each team in that particular game. Other features such as “TeamFTA” and “TeamFTM” were calculated by counting the number of free throws attempted and made by each team in the game, respectively.

### 4.2. Feature Engineering

The primary objective of this paper was to utilize a GNN model to predict basketball game outcomes. To achieve this goal, feature extraction was critical for improving the accuracy of the model. The selection of appropriate features is crucial for accurate prediction, and it appears to be more important for the accuracy than the availability of a large number of games/instances (Bunker et al., 2022) [5]. The manual selection of features based on researchers’ domain expertise can be challenging to interpret, so machine learning algorithms can be employed to output feature importance. The PCA, LASSO, and RF methods were used to extract key variables as input features, reduce the dimension of the input data, and thus improve the model’s performance.

**Figure 4:**
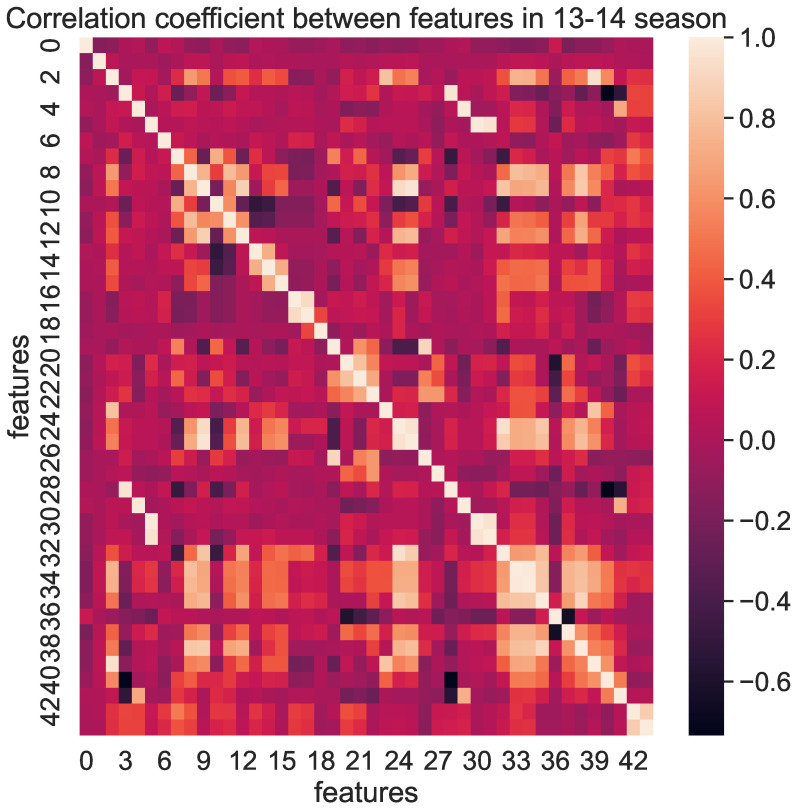
Correlation coefficient heatmap of 44 features in the 13–14 season.

The feature extraction method applied to our GCN model consisted of two steps. The first step involved the correlation coefficient matrix, as illustrated in Figure 4, which displayed the correlation coefficients between the features. A heatmap graphically represented data, using color to depict values. Near the scale of 0, the color indicates that two features are not correlated. As the color gradually becomes lighter, the positive correlation between the two features strengthens. Conversely, as the color darkens, the negative correlation between the two features intensifies. The overall colors suggested that some of the 44 features were highly correlated, indicating redundancy in the feature information. Therefore, performing feature extraction was appropriate and meaningful.

The second step involved the application of three feature extraction methods proposed in this paper. We randomly split the dataset into training, validation, and test sets, using a 70-10-20 ratio. The training set was used to fit the feature extraction models, and then the validation and test sets were transformed using the same models.

The first method was PCA, which is an unsupervised method that maximizes the variance, without using output information. PCA was utilized to reduce the dimension of the features and eliminate correlations among them. To determine the number of principal components n_components, we set this to a contribution rate of 0.95, resulting in the extraction of seven principal components. This step effectively reduced the model complexity, improved its running speed, and eliminated the influence of feature correlation. In subsequent experiments, we utilized a graph-based model, by inputting the seven principal components obtained earlier, in order to examine the model improvement effect.

The LASSO method is a powerful feature extraction technique that is able to reduce variance, select the most relevant features from a potentially large and multicollinear set of predictors, identify redundant predictors, and improve prediction accuracy. After applying LASSO, the coefficients of some features were reduced to zero and these features were excluded from the model. The remaining features with nonzero coefficients were used to build the final model. In our experiment, we used the scikit-learn implementation of the LASSO regression model on the training set to select the most important features, by setting alpha 0.1. To illustrate the application of LASSO, we used data from the NBA 2013–2014 season to predict game outcomes. Our results showed that, out of 44 features, only 12 features were selected by LASSO for prediction. As shown in Table 4, these features included teamEDiff (0.361368), teamPF (−0.037388), teamFTA (0.028293), teamPPS (0.024126), teamDrtg (−0.017633), team3PA (−0.009556), teamBLK% (0.006646), team3P% (0.004803), teamSTL/TO (0.004417), teamDREB% (−0.004153), teamFIC (0.004135), and teamTO (−0.002860). Overall, our findings suggest that LASSO can effectively identify the most important predictors in complex regression models with many potential predictors, and thus improve the prediction accuracy.

To identify the most important features and gain unique insights into their contributions to the prediction task, we employed the random forest (RF) method. In our study, we used the classification and regression tree (CART) algorithm for random forest and the mean decrease impurity method for calculating feature importance. By ranking the features according to their importance, low-importance features could be ignored without adversely affecting the accuracy of the model. This step helped to significantly reduce the noise and data redundancy in the analysis. The results of the RF method, presented in Table 5, provided clear indications of the feature importance, allowing us to reduce the number of features from 44 to 3. Among the top-ranked features, which consistently appeared as the most important across the six seasons, were teamEDiff, teamDrtg, and teamFIC. The extracted features were utilized to train the models, thereby enhancing their overall performance. To ensure the consistency of the feature importance results across the six datasets, we chose the 2013–2014 season as a representative sample. This season was selected because it demonstrated a consistent pattern of change in the feature importance results.

### 4.3. Experimental Results and Comparison

In this section, we present a quantitative analysis of our model, including an evaluation of its prediction accuracy and a comparison of its performance with other models. To construct the graph, we used the open-source fork of Thomas Kipfs’s original GCN model [38] available at [39], and implemented it in Python using Pytorch [40]. The adjacency matrix constructed in Section 3.1 was used to construct the graph that was then fed into the GCN. We split the basketball dataset into 70% for training, 10% for validation, and 20% for testing. Our experiment involved training the GCN model for 500 epochs, with a learning rate of 0.01 and a hidden layer size of 64. To prevent overfitting and improve the model’s generalization, we used a dropout rate of 0.5 during training.

Table 6, Table 7, Table 8 and Table 9 showcase the prediction accuracy of the various graph-based models in predicting basketball game outcomes using the 2012–2018 NBA season dataset. This study found that the graph architectures used in the models were highly effective in predicting the outcomes, with the graph-based models outperforming the previously studied prediction models.

This study also found that combining GCN models with feature extraction resulted in better outcomes than using GCN models alone. Feature extraction was identified as an important factor in achieving better results. We observed that the GCN + RF model achieved the highest average accuracy of 71.54% on all datasets, outperforming the widely used baseline model. The GCN + LASSO model also showed high accuracy levels and outperformed the original GCN model.

However, the study found that GCN + PCA did not yield the desired outcomes. The PCA dimensionality reduction criterion selects principal components that maximize the variance of the original data on the new axis, potentially losing some important information. Moreover, features with small variances are not necessarily unimportant, and such a unique criterion may overlook crucial data. The results suggest that reducing the input space’s dimensionality may have resulted in the loss of vital information necessary for predicting the game’s outcomes.

The aim of this study was to determine the impact of graph-based models on the prediction of basketball game outcomes, and the findings demonstrated that the GCN + RF model achieved the best average performance over the six datasets. The study identified the most critical factors affecting the outcome of NBA games based on the evaluation metrics, providing valuable information for team administrators and coaches, who can utilize this information to improve team playing abilities based on the key factors that affect the winning or losing of a game.

Although adding more features should theoretically result in better prediction results, our study found that the prediction performance improved after extracting important features from the NBA dataset. This emphasizes the significance of appropriate feature extraction for effective prediction. Our results showed that, compared to the original GCN model, the GCN models derived from the RF and LASSO techniques demonstrated improved accuracy. While LASSO outperformed RF in certain seasons, on average, RF showed a slightly better accuracy. However, the GCN + PCA model exhibited a decrease in accuracy.

Furthermore, we compared the prediction results of our proposed model with three models commonly used in previous studies, the decision tree (DT) classifier, the support vector machine (SVM) classifier, and linear regression (LR). Table 6 displays the accuracy of our proposed model and the baseline models in the dataset without feature extraction. The results indicated that the GCN model had a similar predictive power to the DT, SVM, and LR models, and its predictive accuracy was even better when combined with a feature extraction method, as shown in Table 7, Table 8 and Table 9. Our proposed graph-based model was more effective in predicting basketball game outcomes compared to the other models when using LASSO and RF extraction algorithms. It is worth noting that, although Li [28] utilized the same dataset as our study, our proposed model achieved a higher prediction accuracy.

Table 10 presents a performance comparison of the two extraction algorithms, GCN + LASSO and our proposed method (GCN+RF), using 10-fold cross-validation. The data in the table show the accuracy of each method for different seasons, as well as the average accuracy across all seasons. The presented results indicate that our proposed method (GCN+RF) achieved a slightly better average accuracy than GCN + LASSO. Nevertheless, the performance of each method varied across different seasons, indicating that neither method consistently outperformed the other in all situations.

## 5. Conclusions and Discussion

Predicting the outcome of a basketball game is an important but complex task, which relies on several factors, such as a team’s status, the opponent’s situation, and the internal and external environment. In this paper, we propose a graph-based model that takes into account the complex interactions among NBA teams to predict game outcomes. Our model constructs a graph, in which teams are nodes and are connected to their opponents, as well as their own past and future games. These nodes and edges form a message passing network that is trained using a semisupervised GCN model. To improve the prediction accuracy, we combined our model with feature extraction methods. We demonstrated the superiority of our proposed method by comparing it with other prediction studies.

This study used NBA regular season data from the 2012 to 2018 seasons, and the proposed model achieved the highest accuracy compared to other baseline models. Combining the GCN model with feature extraction methods, such as PCA, LASSO, and RF. The RF and LASSO methods improved the model’s accuracy, while the PCA method decreases it. This experiment shows that the one-hop neighbor aggregation in the GCN model gave the best results. Compared to other prediction studies, the proposed model considers the connections between NBA teams and the influence of opponents on the game, providing a new perspective for basketball sports management and performance prediction analysis.

The study has limitations that could be addressed in future research. The homogeneous graph constructed in the current model does not distinguish team nodes from opponent team nodes, and the relationship between nodes is singular. The use of spatiotemporal graphs to represent games on the same date could extend the spatial structure representation of NBA teams and better take into account time information. The category of nodes is a single team representation, and future research could consider adding information such as coaches and players, whose influence on game outcomes is crucial. Further research could apply the proposed model to other basketball leagues, use richer game data, find more suitable feature information and extraction methods, and adjust the number of layers and aggregation methods of the GNN, to improve the accuracy of the model in predicting game outcomes.

## Figures and Tables

**Table 1:** Studies in the literature on predicting the outcome of basketball games.

Author(s)	Year	Amount of Data	Number of Features	Dataset	Model	Success Rate
Loeffelholz et al. [22]	2009	620-Training, 30-Test	11	NBA 2007–2008	FFNN	74.33
Zdravevski and Kulakov [23]	2009	50%-Training, 50%-Test	10	NBA 2consecutive seasons	Logistic Regression	72.78
Miljkovic et al. [24]	2010	778 games	32	NBA 2009–2010	Naive Bayes	67
Cao [25]	2012	80%-Training, 20%-Test	46	NBA 2005–2011	Simple Logistic Regression	69.67
Lin, J. et al. [26]	2014	85%-Training, 15%-Test	17	NBA 1991–1998	Random Forests	65
Tran, T. [27]	2016	-	15	NBA 1985–2015	Dependent Probabilistic Matrix Factorization	72.1
Li, Y. et al. [13]	2019	80%-Training, 20%-Test	10	NBA 2011–2016	DEA	73.95
Horvat, T. et al. [17]	2020	11,578 games	10	NBA 2009–2018	K-NN	60
Li [28]	2020	7380 games	14	NBA 2012–2018	Linear Regression	67.24
Ozkan I A. [18]	2020	240 games	9	Turkish Basketball Super League 2015–2016	CNFS	79.2
ÇENE E. [29]	2022	70%-Training, 30%-Test	-	EuroLeague 2016–2017, 2020–2021	Logistic Regression, SVM, ANN	84
Osken C and Onay C. [30]	2022	-	49	NBA 2012–2018	ANN	76

**Table 2:** Studies in the literature on using GNN to predict the outcome of games.

Author(s)	Year	Amount of Data	Number of Features	Dataset	Model	Success Rate
Aleksandra P. [31]	2021	216,743 games	No feature	52 leagues (Soccer)	GNN	49.62 (multiple leagues), 52.31 (single league)
Xenopoulos P. and Silva C. [32]	2021	4038 games (NFL)	-	NFL 2017 season	GNN	-
Mirzaei A. [33]	2022	21,374 games	Lineup	11 countries and 11 leagues (soccer)	GNN	50.36
Bisberg A J and Ferrara E. [34]	2022	LPL-Training, LCK-Validation, LCS-Test	5	LPL, LCK and LCS	GCN	61.9

**Table 3:** The 44 feature definitions and specific descriptions.

Features	Description	Features	Description
teamLoc	Identifies whether team is home or visitor	teamDayOff	Number of days since last game played by team
teamAST	Assists made by team	teamTO	Turnovers made by team
teamSTL	Steals made by team	teamBLK	Blocks made by team
teamPF	Personal fouls made by team	teamFGA	Field goal attempts made by team
teamFGM	Field goal shots made by team	teamFG%	Field goal percentage made by team
team2PA	Two-point attempts made by team	team2PM	Two-point shots made by team
team2P%	Two-point percentage made by team	team3PA	Three-point attempts made by team
team3PM	Three-point shots made by team	team3P%	Three-point percentage made by team
teamFTA	Free throw attempts made by team	teamFTM	Free throw shots made by team
teamFT%	Free throw percentage made by team	teamORB	Offensive rebounds made by team
teamDRB	Defensive rebounds made by team	teamTRB	Total rebounds made by team
teamTREB%	Total rebound percent by team	teamASST%	Assisted field goal percent by team
teamTS%	True shooting percentage by team	teamEFG%	Effective field goal percent by team
teamOREB%	Offensive rebound percent by team	teamDREB%	Defensive rebound percent by team
teamTO%	Turnover percentage by team	teamSTL%	Steal percentage by team
teamBLK%	Block percentage by team	teamBLKR	Block rate by team
teamPPS	Points per shot by team	teamFIC	Floor impact counter for team
teamFIC40	Floor impact counter by team per 40 min	teamOrtg	Offensive rating for team
teamDrtg	Defensive rating for team	teamEDiff	Efficiency differential for team
teamPlay%	Play percentage for team	teamAR	Assist rate for team
teamAST/TO	Assist to turnover ratio for team	teamSTL/TO	Steal to turnover ratio for team
poss	Total team possessions	pace	Pace per game duration

**Table 4:** The 44 feature coefficients calculated by the LASSO algorithm from the NBA 2013–2014 season.

Features	Score	Features	Score	Features	Score
teamEDiff	0.361368	teamDRB	0.000000	teamFIC40	0.000000
teamFTA	0.028293	teamFGM	0.000000	teamTREB%	0.000000
teamPPS	0.024126	teamFGA	0.000000	teamBLKR	0.000000
teamBLK%	0.006646	teamBLK	0.000000	teamTO%	0.000000
team3P%	0.004803	teamSTL	0.000000	teamOREB%	0.000000
teamSTL/TO	0.004417	teamAST	0.000000	teamEFG%	0.000000
teamFIC	0.004135	teamDayOff	0.000000	teamTS%	0.000000
teamFT%	0.000000	teamORB	0.000000	teamASST%	0.000000
teamFTM	0.000000	teamTRB	0.000000	teamLoc	0.000000
team3PM	0.000000	poss	0.000000	teamTO	−0.002860
team2P%	0.000000	teamSTL%	0.000000	teamDREB%	−0.004153
team2PM	0.000000	teamAST/TO	0.000000	team3PA	−0.009556
team2PA	0.000000	teamAR	0.000000	teamDrtg	−0.017633
pace	0.000000	teamPlay%	0.000000	teamPF	−0.037388
teamFG%	0.000000	teamOrtg	0.000000	-	-

**Table 5:** The 44 features importance ranking based on random forest from the NBA 2013–2014 season.

Features	Score	Features	Score	Features	Score
teamEDiff	0.3846	teamFG%	0.0097	teamPF	0.0038
teamDrtg	0.1163	teamSTL%	0.0092	team2PA	0.0037
teamFIC	0.1018	team3PM	0.0079	poss	0.0033
teamPPS	0.0602	teamAST	0.0069	teamDREB%	0.0033
teamOrtg	0.0438	teamBLK%	0.0057	team2PM	0.0032
teamPlay%	0.0331	teamSTL/TO	0.0057	teamFGA	0.0030
teamEFG%	0.0252	teamFGM	0.0054	teamORB	0.0027
teamTREB%	0.0187	teamAST/TO	0.0051	teamBLK	0.0021
teamTRB	0.0156	teamBLKR	0.0050	teamFTA	0.0018
teamDRB	0.0155	teamFT%	0.0044	pace	0.0017
teamAR	0.0154	teamFTM	0.0042	teamTO	0.0012
team3P%	0.0152	teamTO%	0.0042	teamLoc	0.0007
teamTS%	0.0145	team3PA	0.0040	teamDayOff	0.0007
team2P%	0.0132	teamOREB%	0.0038	teamSTL	0.0007
teamFIC40	0.0101	teamASST%	0.0038	-	-

**Table 6:** Comparison of the prediction performance of GCN and baseline models without feature extraction.

Season	DT Classifier	SVM Classifier	LR	GCN
2012–2013	0.6728	0.7114	0.7029	0.6850
2013–2014	0.6789	0.6965	0.6887	0.6790
2014–2015	0.6707	0.7439	0.7002	0.6900
2015–2016	0.6890	0.6748	0.6820	0.6080
2016–2017	0.6992	0.6585	0.7012	0.6690
2017–2018	0.6829	0.6707	0.6992	0.6830
Average	0.6823	0.6926	0.6957	0.6690

**Table 7:** Comparison of the prediction performance of GCN and baseline models using PCA feature extraction.

Season	DT + PCA	SVM + PCA	LR + PCA	GCN + PCA
2012–2013	0.6087	0.6343	0.6250	0.4960
2013–2014	0.6002	0.6239	0.6190	0.4970
2014–2015	0.5892	0.6022	0.6210	0.4960
2015–2016	0.6103	0.6105	0.6080	0.5107
2016–2017	0.6220	0.6221	0.6102	0.5200
2017–2018	0.6120	0.6342	0.6330	0.5240
Average	0.6071	0.6212	0.6194	0.5073

**Table 8:** Comparison of the prediction performance of GCN and baseline models using LASSO feature extraction.

Season	DT + LASSO	SVM + LASSO	LR + LASSO	GCN + LASSO
2012–2013	0.6928	0.7322	0.7250	0.7295
2013–2014	0.7012	0.6820	0.7009	0.7024
2014–2015	0.7144	0.7023	0.7122	0.7285
2015–2016	0.6912	0.6988	0.6922	0.6829
2016–2017	0.7008	0.7102	0.6820	0.6932
2017–2018	0.7201	0.6979	0.7030	0.7053
Average	0.7034	0.7039	0.7026	0.7070

**Table 9:** Comparison of the prediction performance of GCN and baseline models using random forest feature extraction.

Season	DT + RF	SVM + RF	LR + RF	Our Proposed Method (GCN + RF)
2012–2013	0.7112	0.7152	0.7090	0.7215
2013–2014	0.7022	0.7220	0.7080	0.7154
2014–2015	0.7209	0.7090	0.7201	0.7378
2015–2016	0.7030	0.7113	0.7152	0.7073
2016–2017	0.7103	0.7098	0.7168	0.7154
2017–2018	0.7022	0.6889	0.7012	0.6951
Average	0.7083	0.7094	0.7117	0.7154

**Table 10:** Performance comparison of GCN using two extraction algorithms with 10-Fold Cross-Validation.

Season	GCN + LASSO	Our Proposed Method (GCN + RF)
2012–2013	0.7128	0.7295
2013–2014	0.6890	0.7105
2014–2015	0.6989	0.7285
2015–2016	0.7003	0.6829
2016–2017	0.7054	0.6879
2017–2018	0.6981	0.7106
Average	0.7008	0.7083

## Data Availability

Publicly available datasets were analyzed in this study. The data can be found here: https://www.kaggle.com/datasets/pablote/nba-enhanced-stats, accessed on 9 September 2022.

## Abbreviations

The following abbreviations are used in this manuscript:

ML	Machine Learning
NBA	National Basketball Association
GNN	Graph Neural Networks
GCN	Graph Convolution Network
RF	Random Forest
FFNN	Feed-Forward Neural Network
SVM	Support Vector Machines
ANN	Artificial Neural Networks
DT	Decision Tree
LR	Linear Regression
PCA	Principal Component Analysis
LASSO	Least Absolute Shrinkage and Selection Operator
